# Dataset on the synthesis, characterization, and application of MIL-53(Al)@Biomass hybrid for efficient oxytetracycline elimination via adsorption and photocatalysis

**DOI:** 10.1016/j.dib.2025.111628

**Published:** 2025-05-07

**Authors:** Kevin Jhon Fernández-Andrade, Alex Ariel Fernández-Andrade, Luis E. Arteaga-Pérez, Joan Manuel Rodriguez-Diaz

**Affiliations:** a Facultad de Posgrado, Universidad Técnica de Manabí, Urbina Avenue and Che Guevara, 130105, Portoviejo, Ecuador; b Laboratory of Gas Chromatography and Analytical Pyrolysis, Fac. of Engineering, Universidad del Bío-Bío, Concepción, Chile; c Laboratorio de Análisis Químicos y Biotecnológicos, Instituto de Investigación, UTM, 130105, Portoviejo, Ecuador; d Laboratory of Thermal and Catalytic Processes (LPTC), Wood Engineering Department, University of Bío-Bío, Concepción, 4030000, Chile; e Department of Chemical Engineering, Faculty of Engineering, Universidad de Concepcion, Chile; f Departamento de Procesos Químicos, Biotecnología y Alimentos, Facultad de Ingenierías y Ciencias Aplicadas, Universidad Técnica de Manabí, 130105, Portoviejo, Ecuador

**Keywords:** Antibiotics, Adsorption, Metal-organic framework, Biomass adsorbent, Environmental photocatalysis

## Abstract

Oxytetracycline (OTC) is one of the most used broad-spectrum antibiotics for treating both humans and livestock infections. However, when improperly disposed of, OTC poses a significant risk to water bodies due to its inhibitory effect on water nitrification and its impact on the microbiota. Therefore, various treatments are being investigated to find a sustainable solution for removing oxytetracycline from water. In this sense, adsorption and advanced oxidation processes (AOP) over structured catalytic materials have drawn great attention for their efficiency and selectivity. Important concerns regarding these technical applications are catalyst stability and the proper disposal of adsorbents or catalysts following treatment cycles. Moreover, the elucidation of the underlaying kinetic and thermodynamic mechanisms governing these processes are also of paramount importance for process design and optimization. Accordingly, here we provide a complete dataset on the synthesis and characterization of a novel MOF@Biomass layered hybrid (MIL-53(Al)@RH) for the removal of OTC from water bodies via adsorption and LED-driven photocatalysis. The MIL-53(Al)@RH was synthetized by a microwave-assisted solvothermal method enabling the in-situ hybridization of MIL-53(Al) on rice husk (RH) as a biomass support. The dataset contains information on the physicochemical properties of pristine materials (MIL-53(Al) and rice husk), MIL-53(Al)@RH, and a Carbon-Al obtained via pyrolysis of the saturated MIL-53(Al)@RH (after adsorption). Moreover, data on BATCH adsorption experiments and photocatalytic elimination of oxytetracycline with MIL-53(Al)@RH is also provided. Adsorption data include information on the effect of MIL-53(Al)@RH dosage, solution pH, and kinetics measurements at different initial concentrations of OTC. In addition, data on the photocatalytic activity of MIL-53(Al)@RH is included for different oxidant doses (H_2_O_2_), OTC concentrations using a Led-light source.

Specifications Table

**Table:** 

Subject	Chemical Engineering.
Specific subject area	Adsorption and Photocatalysis
Type of data	Figures and Tables. Raw and processed
Data collection	OTC concentration was measured by HPLC (Accela Thermo Scientific) interfaced with a quaternary pump and a photo diode array (PDA) using a C18 (Thermo Hypersil GOLD 25,005–104,630) column. Kinetic and thermodynamic interpretation of the data was performed in excel spreadsheets. The MOF@Biomass hybrid was characterized by multiple techniques such as N_2_ physisorption (Gemini VII 2390 series), FTIR-ATR spectrum was recorded between 400 and 4000 cm^-1^ in a Nicolet is20 spectrometer, and by SEM in a JEOL JSM-6610LV microscope operated at 7 kV and 3000 X magnification. Micropyrolysis experiments of saturated MIL-53@RH, were performed in a CDS5200 unit connected to a GC–MS (Clarus 690, QS8, Perkin Elmer).
Data source location	Data was collected in the Laboratorio de Análisis Químicos y Biotecnológicos, Instituto de Investigación, Universidad Técnica de Manabí, S/N, Avenida Urbina y Che Guevara, Portoviejo, 130,104, Ecuador (−1.046156, −80.455019).
Data accessibility	Repository name: Mendeley Data Data identification number: 10.17632/7byx73sk82.3 Direct URL to data: https://data.mendeley.com/datasets/7byx73sk82/3 Data is freely available at above link
Related research article	K.J. Fernández-Andrade, A.A. Fernández-Andrade, B.F. Rivadeneira-Mendoza, L.A. Zambrano-Intriago, L.E. Arteaga-Pérez, J.M.R. Díaz, Highly efficient MIL-53(Al)@Biomass hybrid for oxytetracycline elimination: Adsorption, LED-induced photocatalysis and pyrolytic recycling, Journal of Env. Chem. Eng. 12 (2024) 114,628. https://doi.org/10.1016/j.jece.2024.114628.

## 1 Value of the Data

• This dataset demonstrates the effectiveness of a MIL-53(Al)@RH hybrid in removing recalcitrant antibiotics, specifically oxytetracycline, through both synchronous and asynchronous adsorption-photocatalysis processes.
• The data is of paramount importance to researchers and engineers who are in pursuit of circular economy solutions for revalorizing wasted adsorbents as it provides a method for using saturated MIL-53(Al)@RH hybrid as templates for new carbon-based adsorbents.
• The kinetic analysis of adsorption and photocatalysis in this dataset is essential for the optimization of the design of adsorbents and catalysts, the prediction of process efficiency, and the identification of rate-limiting steps.
• The thermodynamic insights obtained from isotherm fitting contribute to a more comprehensive understanding of process mechanisms and offer detailed information on the molecular interactions between contaminants and MIL-53(Al)@RH hybrids.
• The data provided in this study can be useful for scaling up water treatment systems using novel adsorbents or photocatalysts such as MOF@Biomass hybrids to remove recalcitrant and complex pollutants such as antibiotics from different water bodies.

## 2 Background

Oxytetracycline is a widely used broad-spectrum antibiotic; therefore, it is one of the most concerning forms of contamination for water exposed to anthropogenic activities [[1], [2], [3]]. The OTC is stable in slightly acidic aquatic environments at low temperatures; yet its presence at trace amounts prevents water nitrification and significantly affects the microbial ecology [3]. Furthermore, OTC is highly resistant to standard wastewater treatments, thus it persists in ecosystems for extended periods of time. As a result, there is an urgent need to develop new treatment technologies such as adsorption and photocatalysis to reduce OTC contamination [4] which have demonstrated efficacy against other pollutants like dyes [5,6]. In this context, the use of metalorganic frameworks (MOFs) such as MIL-53(Al) has received considerable attention, as these structured materials show activity for adsorption and photocatalytic water treatments. However, virgin MOFs have low mechanical performance, thus they must be functionalized with other templates before being deployed at plant scales. We present a comprehensive dataset that includes chemical characterization of MIL-53(Al)@RH hybrid utilizing spectroscopy, microscopy, and chemical analysis. Furthermore, the dataset includes a thorough investigation of the application of MIL-53(Al)@RH hybrid for synchronous and asynchronous OTC adsorption and photocatalytic removal. Finally, we have included information on the characterization and adsorption performance of a carbonaceous material obtained by pyrolyzing the saturated MIL-53(Al)@Biomass hybrid. This dataset is related to recently published research in which we reveal mechanistic insights into the removal of OTC using MIL-53(Al)@RH hybrid via adsorption and photocatalysis. In that work, we also offered a complete investigation of the MIL-53(Al)@RH hybrid's structure-activity performance, as well as its role in lowering water toxicity following treatment. This paper provides the raw data that supports the interpretations and findings discussed in previously published papers related to adsorption and photocatalysis.

## 3 Data Description

The dataset presented in this study covers results on the physicochemical characterization and performance of a new MIL-53(Al)@RH layered hybrid for OTC removal from water bodies using asynchronous and synchronic adsorption and LED-driven photocatalysis. MIL-53(Al)@RH was synthesized using a microwave-assisted solvothermal technique, allowing for in-situ hybridization of MIL-53(Al) with a biomass template (rice husk). Several complementary techniques were used to characterize the MIL-53(Al)@RH hybrid, including Fourier Transformed Infrared Spectroscopy coupled with attenuated total reflection accessory (FTIR-ATR), UV–Visible Diffuse Reflectance Spectroscopy (UV–Vis DRS), X-ray diffraction (XRD), scanning electron microscopy with energy dispersive X-ray spectroscopy (SEM-EDX), N_2_-physisorption at 77 K, and The pH of the point of zero charge (pH_pzc_). Furthermore, the dataset contains an investigation of the pyrolysis conditions used to treat the saturated MIL-53(Al)@RH hybrid following numerous adsorption-reaction cycles. This investigation was carried out using analytical pyrolysis coupled to mass spectrometry (Py-GC–MS), hence crude mass spectra are given alongside the files for product identification.

**Fig. 1:**
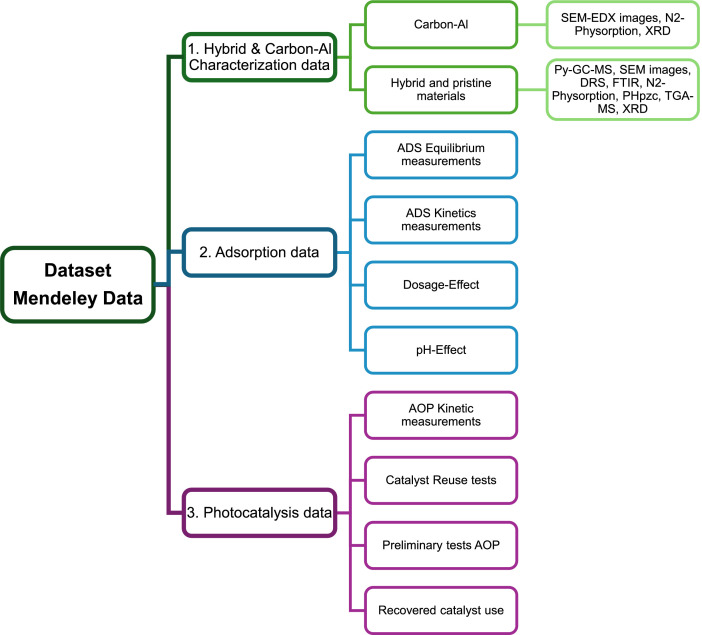
Data ordering provided in the Data Mendeley repository.

The data can be openly accessed through a Mendeley Data repository via the link provided in the data accessibility section. A brief overview of the data, the access routes, and technical details about the dataset is presented in the description available in the Mendeley Repository, while a navigation map describing the folders ordering and content is provided in Fig. 1. The files are accessible for download or can be viewed directly in the repository's previews. Finally, the data files are included in several formats, thus the reader can use different visualization or data-treatment software to manage the information.

**Folder 1:** Hybrid and Carbon-Al characterization data: Includes comprehensive characterisation data for virgin materials, MIL-53(Al)@RH, and recovered Carbon-Al following hybrid saturation. This work presents the FTIR-ATR spectra, XRD patterns, N_2_ adsorption-desorption isotherms, pore size distributions, BET calculations, and BJH model results. In addition, the pH_pzc_ and UV–Vis DRS profile, scanning electron microscopy (SEM) pictures, and a Py-GC–MS study on the decomposition of the catalyst are reported. Further information about the interpretation of the characterization in our research paper [7]. The main conclusions derived from this dataset indicate that the adsorptive and photocatalytic properties of the hybrid material are defined by a synergistic effect of the structural and chemical properties, of the MOF and the biomass precursor (rice husk).

**Fig. 2:**
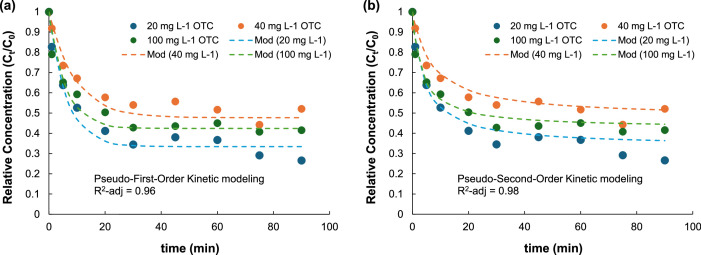
Results of the adsorption kinetic assays and modelling for Initial concentration of OTC (C_0_) = 20 mg l^-1^, 40 mg l^-1^, and 100 mg l^-1^. Conditions: Catalyst = 0.55 *g* *L*^−1^, pH = 4.24 – 4.86, stirring = 300 rpm, Temperature = 25 °C. (a) Pseudo-first-order model fitting (b) Pseudo-second-order model fitting.

**Folder 2:** Adsorption data: This folder summarizes the results of the adsorption experiments including pH and MIL-53(Al)@RH doses, kinetic and equilibrium measurements. The results and calculations are implemented in excel spreadsheets. Examples of these data are provided in Table 1 and Fig. 1, Fig. 2. More details on the modelling results isotherms adjusts and mechanistic insights are provided in a related research paper [8].

**Table 1:** Results from adsorption experiments using MIL-53(Al)@RH hybrid. Measurements under batch conditions, C_0_ = 20 mg l^-1^, Catalyst = 0.55 g l^-1^, pH = 4.23 – 4.75.

Time	C_t_ (mg l^-1^)	% Removal	Q_t_ (mg g^-1^)	C_t_/C_0_
0	24.88	0.00 %	0	1
1	20.58	17.29 %	7.82	0.82
5	15.87	36.20 %	16.38	0.63
10	13.11	47.30 %	21.40	0.52
20	10.25	58.80 %	26.60	0.41
30	8.58	65.49 %	29.63	0.34
45	9.48	61.91 %	28.01	0.38
60	9.14	63.24 %	28.61	0.36
75	7.25	70.85 %	32.05	0.29
90	6.62	73.40 %	33.21	0.26
Replicate-1				
0	24.89	0.00 %	0	1
1	20.88	16.12 %	7.30	0.84
5	14.67	41.06 %	18.58	0.59
10	14.32	42.46 %	19.21	0.58
20	10.18	59.09 %	26.74	0.41
30	8.97	63.97 %	28.95	0.36
45	8.10	67.46 %	30.53	0.33
60	8.97	63.95 %	28.94	0.36
75	8.62	65.37 %	29.58	0.35
90	8.43	66.14 %	29.93	0.34
Replicate-2				
0	24.89	0.00 %	0	1
1	19.35	22.25 %	10.07	0.78
5	15.44	37.97 %	17.18	0.62
10	13.14	47.19 %	21.35	0.53
20	11.66	53.15 %	24.05	0.47
30	10.51	57.79 %	26.15	0.42
45	9.26	62.79 %	28.41	0.37
60	7.25	70.89 %	32.08	0.29
75	9.39	62.29 %	28.19	0.38
90	9.91	60.20 %	27.24	0.40

C_0_ is the initial concentration of OTC, C_t_ is the OTC concentration measured at time = *t*, % Removal represents the MIL-53(Al)@RH relative removal capacity, Qt is the adsorption capacity.

**Folder 3:** Photocatalysis data: This folder encloses the results of photocatalytic elimination of OTC using MIL-53(Al)@RH hybrid as a photocatalyst. Readers will find results from the measurements of preliminary tests and kinetics, catalyst reuse cycles, and application of the carbon structure derived from MIL-53(Al)@RH after saturation and pyrolysis.

**Fig. 3:**
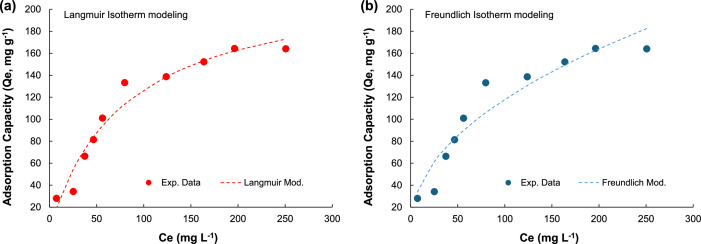
Experimental and fitted adsorption isotherms. (a) Langmuir´s isotherm and (b) Freundlich´s isotherm. Conditions: Catalyst = 0.55 *g* *L*^−1^, pH = 4.24 – 4.86, stirring = 300 rpm, Temperature = 25 °C, Equilibrium time = 45 min.

**Fig. 4:**
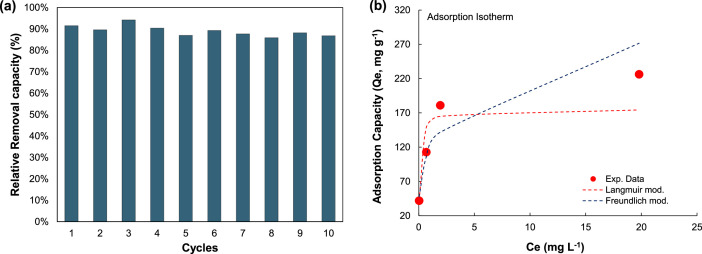
Results of MIL-53(Al)@RH operational cycles (adsorption-photocatalysis) and adsorption performance of carbonized MIL-53(Al)@RH after saturation: (a) Catalyst reuse tests at C0 = 60 mg l^-1^, (b) Adsorption at equilibrium and isotherms fitting. Conditions: Catalyst = 0.55 *g* *L*^−1^, pH = 4.24 – 4.86, C0 = 20, 60, 100, 140, 220 mg L^–1^, Equilibrium adsorption time = 45 min, C0H2O2 = 170 mg *L*^−1^, LED light exposure time = 20 min, stirring = 300 rpm, temperature 25 °C.

The results of adsorption and photocatalysis demonstrated that the hybrid's remarkable efficiency in removing up to 90 % of the drug from water under extreme conditions (200 mg l^-1^ OTC) was linked to the surface coverage and limited by the oxidant/pollutant ratio. The synchronous adsorption-photocatalysis process showed that water treatment effectively eliminated the drug and significantly lowered the toxicity of the contaminated water to below LD50 levels (Fig. 3, Fig. 4).

## 4 Experimental Design, Materials and Methods

### 4.1 Materials

Sodium hydroxide (NaOH 99 %, CAS: 1310–73–2, Merck USA), hydrogen peroxide (H_2_O_2_ 30 %, CAS: 7732–84–1, Fisher USA), ultrapure water (18.2 MΩ·cm), and hydrochloric acid (HCl 35 %, CAS: 7647–01–0, Merck USA). Additionally, the chemicals 1,4-Benzenedicarboxylic acid (1,4-BDC, CAS: 100–21–0), Aluminium Chloride Hexahydrate (AlCl3·6H2O, CAS: 7784–13–6) and N–N-Dimethylformamide (DMF, CAS: 68–12–2) oxytetracycline hydrochloride (OTC, CAS: 2058–46–0) were acquired from Sigma Aldrich USA.

### 4.2 Synthesis of mil-53@rh hybrid

**Fig. 5:**
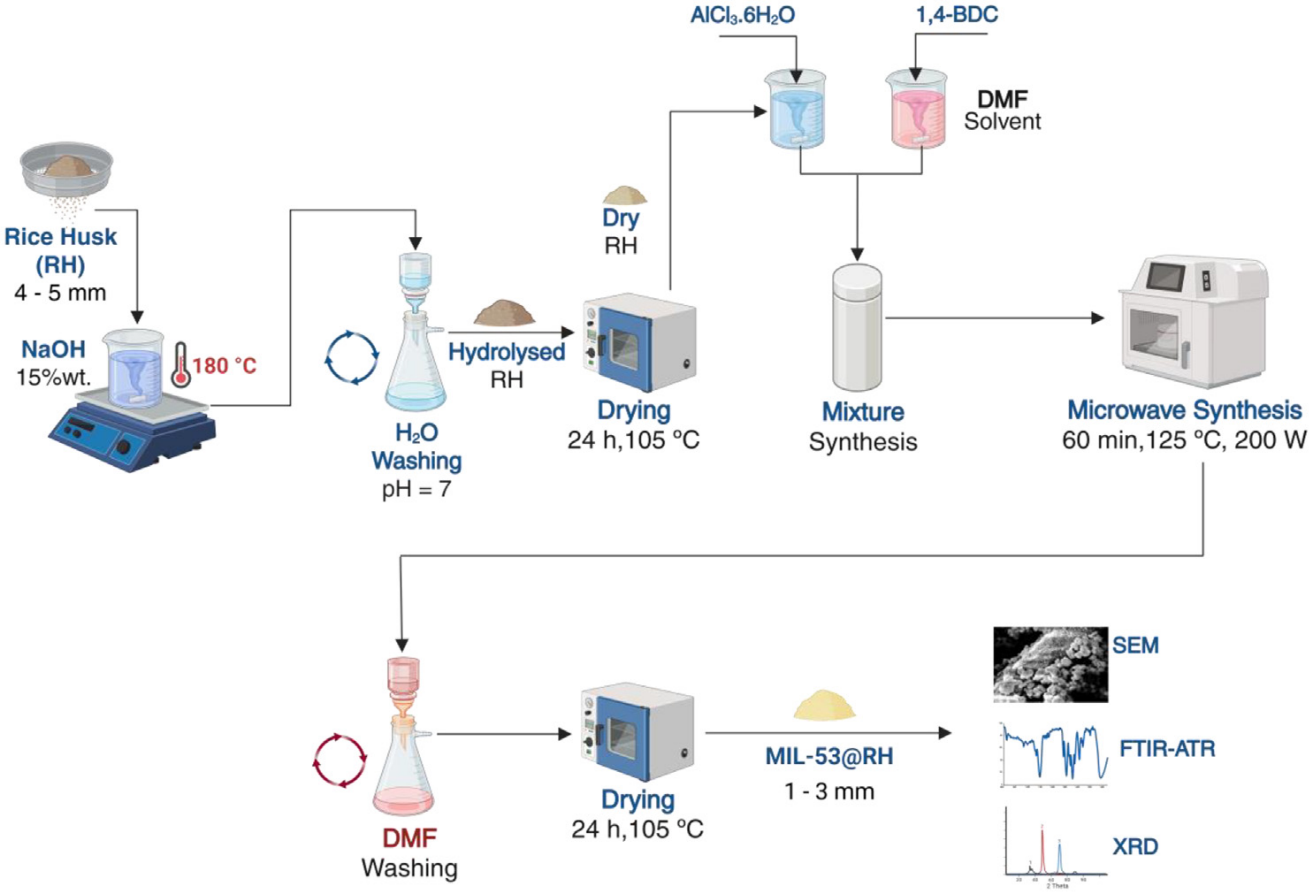
Experimental protocol for the synthesis of MIL-53@Hybrid. Created with BioRender.com.

The rice husk was acquired from a local producer and pre-treated in accordance with our group's prior publication [9,10]. Briefly, RH was milled and sieved to 4–5 mm particle size. The RH was then rinsed with distilled water and dried at 105 °C for 24 hours (Fig. 5). Before hybridization with the MOF, the RH was activated by partial hydrolysis. To activate the RH, we placed 30 g of dry RH in a beaker containing a 15 % wt. NaOH solution for thermo-alkali hydrolysis. The RH—NaOH slurry was then covered with aluminium foil, heated to 180 °C, and agitated at 300 rpm for 4 hours. The resultant RH was washed until neutralized with distilled water before drying at 105 °C for 24 hours.

In a beaker, 16 mmol of 1,4-BDC was dissolved in 60 mL of DMF using magnetic stirring at 300 rpm until a clear and crystalline solution (Solution A) was formed. In another beaker, 15 mmol of AlCl_3_ ⋅6H2O was dissolved in 6 mL of ultrapure water. In the same beaker as the aluminium salt solution, 2 g of RH was added and magnetically stirred for 15 min (Solution B). Then, Solution B was mixed with Solution A. The reagent solutions were mixed into a crystalline mixture and placed in a Teflon tube. The tube was then placed in a CEM MARS 6 microwave oven for 60 min at 125 °C and 200 W power for the reaction. Three DMF washes removed the excess 1,4-BDC from the solid, followed by ultrapure water washes to remove the DMF from the hybrid. Finally, the hybrid was dried at 105 °C for 24 h in static air. The same technique, but without the inclusion of RH, was used to synthesize pure MIL-53(Al).

### 4.3 Characterization of mil-53@rh hybrid

The MIL-53@RH hybrid was characterized by several techniques such as:

**Scanning Electron Microscopy (SEM):** Images were recorded in a JEOL JSM-6610LV (Tokio, Japan), microscope at 7 kV and 3000 X magnification.

**Nitrogen Physisorption:** N_2_ adsorption and desorption isotherms were recorded on a Micromeritics Gemini VII 2390 series (Massachusetts, USDA) to analyze textural properties (specific surface and pore size distribution). The samples underwent a 12 h degassing at 110 °C, and the resulting isotherms were fitted to by Brunauer-Emmett-Teller (BET) and Barrett-Joyner-Halenda (BJH) models.

**X-Ray Diffraction (XRD):** X-Ray Diffraction (XRD) patterns were obtained with a Rigaku X-Ray Diffractometer Smartlab (Tokio, Japan), with Theta-Theta Bragg-Brentano geometry, a D/teX model Ultra 250 solid-state detector, and Cu radiation (1541 Å) at 40 kV and 30 mA.

**Fourier Transform Infrared Spectroscopy (FTIR):** Fourier Transform Infrared Spectra (FTIR) were collected in a Nicolet is20 spectrometer (Thermo Scientific, USA) equipped with an Attenuated Total Reflectance (Quest accessory (ATR) using a Ge crystal with a 0.65 µm penetration depth. The spectra were averaged from 32 scans recorded between 600 and 4000 cm^−1^ at a resolution of 4 cm^−1^.

**UV–Vis Diffuse Reflectance Spectroscopy (UV–Vis DRS):** The analysis was carried out with a Lambda UV–vis-NIR Spectrometer (PerkinElmer, USA) equipped with a DRS accessory to measure the absorption of UV–Vis light and to determine the optical properties of the materials by the Tauc plot method. The test conditions include a scanning wavelength range from 200 nm to 1000 nm with a step size of 5 nm. Bario sulphate served as the reference substance.

**Point of Zero Charge (pH_pzc_):** The pH of the point of zero charge point (pH_pzc_) was determined by a method detailed in a prior study [10]. This involves putting flasks containing ultrapure water with a modified pH ranging from 2 to 12 at intervals of 1. Each flask received 0.1 g of material and was maintained in agitation for 24 hours. The initial and final pH (after 24 h) were measured to construct the curve (pH_0_-pH_f_) vs pH_0_, with the point where the curve intersects zero representing the pH of the point of zero charge.

**Batch adsorption experiments:** Preliminary adsorption tests were performed using 50 mL beakers loaded with 40 mg *L*^−1^ of OTC and varying dosages of MIL-53(Al)@RH between 0.1 and 1.3 g *L*^−1^. The solutions were stirred at 300 rpm and covered with aluminum foil to avoid OTC photodegradation during adsorption (dark experiments). To establish adsorption equilibrium, the measurement time was set at 4 hours [10]. After estimating the adsorbent doses at which Q_e_ reached a plateau, the pH of the solution was tested between 2 and 10 to guarantee that further kinetic measurements were done without influence from doses or pH.

The dosage and pH for OTC adsorption were determined using the equilibrium adsorption capacity (Q_e_, Eq. (1)) and relative removal capacity at the adsorption equilibrium (Eq. (2)):

(1) $$Q_{e}=\frac{\left(C_{\text{OTC}}^{0}-C_{\text{OTC}}^{e}\right)\cdot V}{m}$$

(2) $$\%\text{Removal}=\frac{C_{\text{OTC}}^{0}-C_{\text{OTC}}^{e}}{C_{\text{OTC}}^{0}}\times \;100$$

here, C^0^_OTC_ and C^e^_OTC_ are the initial and equilibrium concentrations (mg *L*^−1^) of OTC, respectively, V is the volume of OTC solution (L), and m is the mass of MIL-53(Al)@RH (g). To determine the values of Qt and %Removal at different times, it is sufficient to replace the equilibrium concentration of OTC with the concentration at time t (C_t_).

Concentration was determined using a high-performance liquid chromatograph (HPLC, Accela Thermo Scientific) with a quaternary pump and a photo diode array (PDA). The analyte was separated using a column C18 (Thermo Scientific model Hypersil GOLD 25,005–104,630) using the technique established by Giler-Molina et al. [11]. The approach involves injecting 10 µL of sample at 354 nm wavelength and 25 °C. The mobile phase, consisting of acetonitrile and acidified water (pH = 2) (80:20), flowed at 900 µL min^-1^.

### 4.4 Thermodynamic and kinetic interpretation of OTC adsorption onto mil-53@rh hybrid

Adsorption kinetics and equilibrium were studied for the pH and MIL-53(Al)@RH dosage established in the preliminary assays described before. The kinetic study was performed during 90 min at 25 °C (sampling every minute), and for a wide range of initial OTC concentrations (20 to 220 mg *L*^−1^). The C^e^_OTC_ was measured after 4 h sampling for each initial concentration. Moreover, the effect of temperature (25, 40 and 60 °C) on the adsorption equilibrium was studied. The available data was applied to estimate adsorption parameters for several kinetic and equilibrium models as those reported in Table 2:

**Table 2:** Equilibrium and kinetic models used to interpret the adsorption data.

Model	Equation	Parameters	Reference
Kinetic models			
Pseudo-First Order	$q_{t}=q_{e}\left(1-e^{-(K_{1}t)}\right)$ (3)	qt: adsorption capacity over time (mg g^-1^) qe: adsorption capacity at equilibrium (mg g^-1^) t: contact time (min) K1: Pseudo-first-order kinetic constant (min^-1^) K2: Pseudo-second-order kinetic constant (g^-1^min^-1^)	[12]
Pseudo-Second Order	$q_{t}=\frac{K_{2}q_{e}^{2}t}{1+K_{2}q_{e}t}$ (4)	[13]	
Elovich	$q_{t}=\left(\frac{1}{\beta }\right)\ln\left(1+\alpha \beta t\right)$ (5)	α: initial adsorption rate (mg g^-1^ min^-1^) β: desorption constant (g mg^-1^)	[14]
Bangham	$q_{t}=K_{b}t^{\alpha }$ (6)	α: adsorption intensity Kb: Bangham constant rate	[15]
Equilibrium models			
Langmuir	$q_{e}=\frac{Q_{\text{max}}^{0}K_{L}C_{\text{OTC}}^{e}}{1+K_{L}C_{\text{OTC}}^{e}}$ (7)	Q0max: maximum adsorption capacity (mg g^-1^) KL: Langmuir constant (L mg^-1^) Ce_OTC_: equilibrium concentration (mg L^-1^)	[16]
Freundlich	$q_{e}=K_{F}C{{}_{\text{OTC}}^{e}}^{1/n}$ (8)	KF: Freundlich constant (L^1/n^ mg^1–1/n^g^-1^) n: surface heterogeneity	[17]
Sips	$q_{e}=\frac{Q_{m}{\left(K_{S}C_{\text{OTC}}^{e}\right)}^{\frac{1}{n}}}{1+{\left(K_{S}C_{\text{OTC}}^{e}\right)}^{\frac{1}{n}}}$ (9)	Ks: Sips constant (L^1/2^g^-1/2^) n: heterogeneity index Qm: maximum adsorption capacity (mg g^-1^)	[18]

### 4.5 Photocatalytic assays and kinetic modeling

The catalytic effect of MIL-53@RH in photochemical decomposition of OTC was investigated under different operational conditions. Individual studies were used to conduct preliminary assays to discover the key variables impacting OTC degrading performance (H_2_O_2_, LED light, and catalyst). A standard 50 W white LED light served as the light source, positioned 10 cm distant from the sample. Experiments were conducted with 50 mL of OTC at several beginning concentrations, including C^0^_OTC_: 20, 40, 60, 80, 100, 140, 180, and 220 mg *L*^−1^. The solutions were treated with MIL-53(Al)@RH (appropriate dosage) in the absence of light and H_2_O_2_ until adsorption equilibrium was reached (4 h). After that, each solution was exposed to LED light with varying H_2_O_2_dose and contact times: C^0^_H2O2_: 130, 170, and 210 mg *L*^−1^; times: 0, 1, 3, 5, 7, 10, 13, 16, 20, 25, and 30 min. All studies were carried out at 298 K with continuous stirring (300 rpm).

### 4.6 Production of carbon-al after mil-53@rh hybrid saturation

**Fig. 6:**
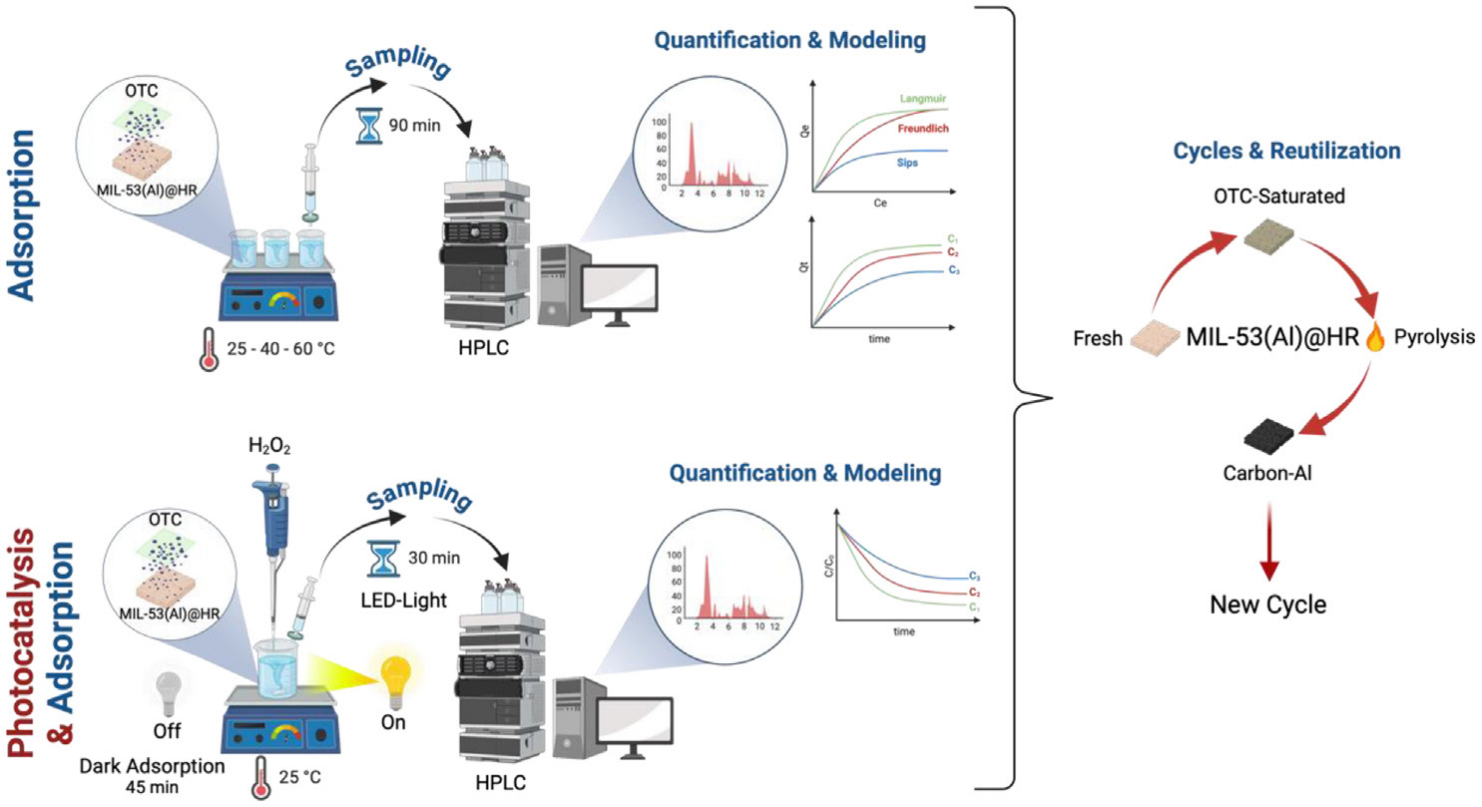
Experimental protocol for the generation, interpretation and application of the dataset. Reproduced from [8] with permission from Elsevier.

To inspect the reusability of wasted MIL-53@RH, a sample of this catalyst was oversaturated with OTC and thermally treated at high temperature. The treatment consisted of a pyrolysis at 993 K, at a constant N_2_ flow rate of 120 mL min^-1^. The heating ramp for this treatment was set at 10 K min^-1^ and the dwell period at 2 hours. This thermal treatment enables the breakdown of the OTC and the formation of a Carbon-Al structure which maintains adsorption capacity. This structure is designed to facilitate a cyclical processing of the depleted catalysts (Fig. 6).

## Limitations

The data gathered were sourced from a laboratory scale and within a batch system functioning in a non-stationary regime. While the data holds significant value for understanding the thermodynamics and kinetics of the adsorption and photocatalytic performance of the MIL-53(Al)@Biomass hybrid, additional analysis will be necessary for effective process design and scale-up.

## Ethics Statement

The authors have thoroughly reviewed and adhered to the ethical guidelines for publishing in Data in Brief. They affirm that the present study does not include any data obtained from social media sites, animal trials, or human participants.

## Credit Author Statement

**Kevin Jhon Fernández-Andrade:** Conceptualization, Methodology, Investigation, Writing - Review & Editing, **Alex Ariel Fernández-Andrade:** Formal analysis, Validation, Writing - Review & Editing, **Luis E. Arteaga-Pérez:** Visualization, Data Curation, Writing - Original Draft, **Joan Manuel Rodríguez Díaz:** Conceptualization, Resources, Supervision, Project administration, Funding acquisition.

## Data Availability

Mendeley DataDataset for oxytetracycline elimination via adsorption and LED-induced photocatalysis using a MIL-53(Al)@Biomass layered hybrid (Original data).
